# Pd nanosheets with their surface coordinated by radioactive iodide as a high-performance theranostic nanoagent for orthotopic hepatocellular carcinoma imaging and cancer therapy†
†Electronic supplementary information (ESI) available. See DOI: 10.1039/c8sc00104a


**DOI:** 10.1039/c8sc00104a

**Published:** 2018-04-12

**Authors:** Mei Chen, Zhide Guo, Qinghua Chen, Jingping Wei, Jingchao Li, Changrong Shi, Duo Xu, Dawang Zhou, Xianzhong Zhang, Nanfeng Zheng

**Affiliations:** a The State Key Laboratory for Physical Chemistry of Solid Surfaces , Collaborative Innovation Center of Chemistry for Energy Materials , National & Local Joint Engineering Research Center of Preparation Technology of Nanomaterials , Department of Chemistry , Xiamen University , Xiamen 361005 , China . Email: nfzheng@xmu.edu.cn; b College of Materials Science and Engineering , Hunan University , Changsha 410082 , China; c Center for Molecular Imaging and Translational Medicine , State Key Laboratory of Molecular Vaccinology and Molecular Diagnostics , School of Public Health , Xiamen University , Xiamen 361102 , China . Email: zhangxzh@xmu.edu.cn; d State Key Laboratory of Cellular Stress Biology , Innovation Center for Cell Signaling Network , School of Life Sciences , Xiamen University , Xiamen , Fujian 361102 , China

## Abstract

Radiolabeled nanoparticles (NPs), taking advantage of nanotechnology and nuclear medicine, have shown attractive potential for cancer diagnosis and therapy. However, the high background signal in the liver and long-term toxic effects of radioisotopes caused by the nonselective accumulation of radiolabeled nanoparticles in organs have become the major challenges. Here, we report a pH-sensitive multifunctional theranostic platform with radiolabeled Pd nanosheets through a simple mixture of ultra-small Pd nanosheets and radioisotopes utilizing the strong adsorption of ^131^I and ^125^I on their surfaces (denoted as ^131^I–Pd–PEG or ^125^I–Pd–PEG). Systematic studies reveal that the labeling efficiency is higher than 98% and the adsorption of radioiodine is more stable in an acidic environment. *In vivo* studies further validate the pH-dependent behavior of this platform and the enhanced retention of radioisotopes in tumors due to the acidic microenvironment. Single photon emission computed tomography (SPECT) images with zero background were successfully achieved in a subcutaneous 4T1 tumor model, an orthotopic LM3 tumor model, and even in a *Mst1/2* double-knockout hepatoma model. Moreover, the application of radiolabeled Pd nanosheets for photoacoustic (PA) imaging, and combined photothermal and radiotherapy was also explored. Therefore, this study provides a simple and efficient strategy to solve the critical high background issue of radiolabeled nanoparticles and shows enormous potential for clinical applications.

## Abstract 2

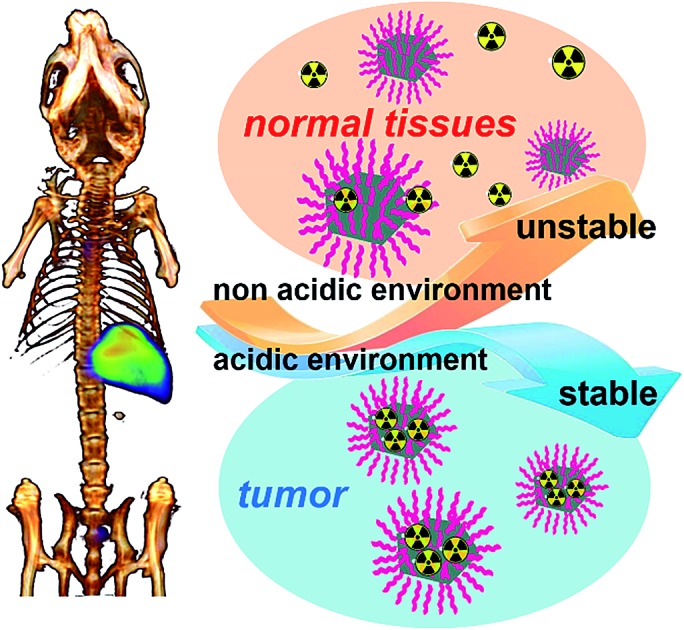
We report a pH-sensitive multifunctional theranostic platform with radiolabeled Pd nanosheets through a simple mixture of ultra-small Pd nanosheets and radioisotopes utilizing the specific adsorption of ^131^I and ^125^I on their surfaces.

## Introduction

Radiolabeled nanoparticles (NPs) for positron emission tomography (PET) and single photon emission computed tomography (SPECT) have received special attention owing to their high sensitivity, deep tissue penetration and improved pharmacokinetics.^1^ In recent decades, various combinations of radioisotopes and nanomaterials have been successfully developed for the early diagnosis of cancer, such as ^64^Cu labeled MoS_2_ nanosheets,^2^^125^I labeled carbon nanotubes,^3^ and ^131^I labeled reduced graphene oxide.^4^ Although prolonged half-life and enhanced accumulation of radiolabeled NPs in tumor tissues can be achieved, they do have several limitations. The background signal caused by high accumulation of NPs in the liver has been the most crucial factor degrading the quality of imaging, even for those modified with active target molecules.1f,^5^ Moreover, the long-term retention of radioisotopes in normal tissues may cause toxic effects *in vivo*. Currently, most studies are focused on the imaging of subcutaneous xenograft tumors, and few reports have demonstrated their potential application in deep tissue imaging especially in orthotopic liver cancer. Therefore, the development of radiolabeled NPs to minimize the background signal and maximize the tumor-to-normal tissue (T/N) ratio is highly desired.

Tumor microenvironment sensitive theranostic agents utilizing the difference between tumor and normal tissue have been used to improve the T/N ratio of tumors.1b,^6^ In particular, pH-responsiveness is most frequently used.^7^ An acidic microenvironment (pH = 6.5–6.8) is a typical feature of the solid tumor extracellular environment, while the pH value of blood and normal tissues is neutral (pH = 7.4).^8^ Recently, great improvement in tumor imaging and therapy has been made by using pH-sensitive nanoplatforms.^9^ Considering the high background of radionuclide imaging, construction of pH-sensitive radiolabeled nanoparticles may provide a new strategy for imaging of intrahepatic anatomy and precise localization.

Halide ions have played important roles in the shape-controlled synthesis of noble metal nanocrystals because of their strong adsorption on specific facets.^10^ By introducing halide ions (*e.g.*, Br^–^), uniform hexagonal Pd nanosheets could be synthesized with sizes ranging from sub-5 nm to 120 nm.^11^ And the interaction of halide ions with Pd follows the order of I^–^, Br^–^, and Cl^–^.^12^ Meanwhile, radioactive iodine isotopes have attracted great interest due to their widespread application in molecular imaging and nuclear medicine. For example, ^123/125/131^I has been, in various forms, the mainstay of SPECT imaging and ^124^I is used for PET imaging. ^131^I with β emission has contributed more than other radionuclides to radiotherapy in nuclear medicine.^3^,^13^ Therefore, the combination of Pd nanomaterials with radioiodine may create new opportunities for cancer theranostics.

**Scheme 1:**
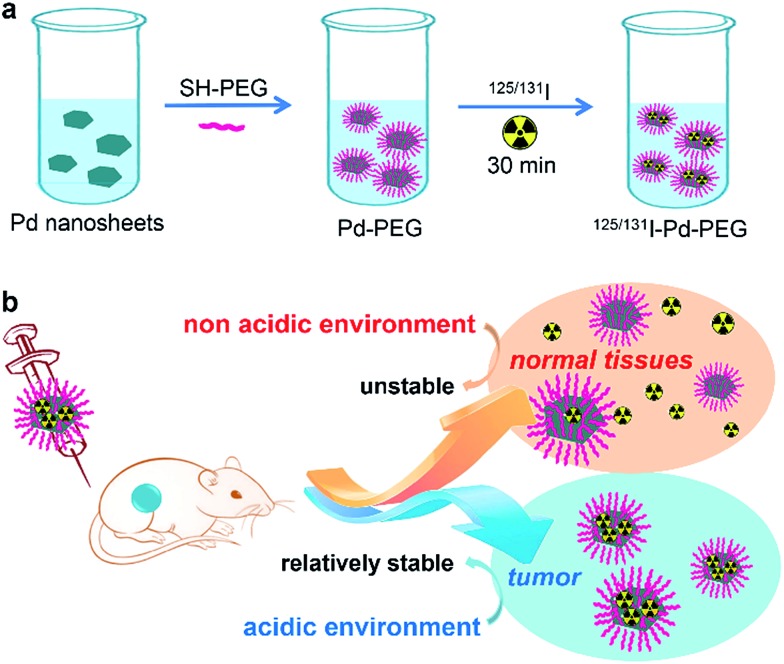
(a) Labeling procedure of radioiodine on the surface of Pd nanosheets. (b) *In vivo* pH-dependent behavior of radiolabeled Pd nanosheets.

Ultra-small Pd nanosheets have been reported as one kind of photothermal agent exhibiting excellent biocompatibility, high photothermal conversion efficiency and urine clearance.^14^ Herein, we report on a pH-sensitive multifunctional theranostic platform based on radiolabeled Pd nanosheets, obtained through a simple mixing of ultra-small Pd nanosheets with coordinating ^125/131^I (denoted as ^131^I–Pd–PEG or ^125^I–Pd–PEG) (Scheme 1a). Interestingly, the adsorption of radioiodine on Pd nanosheets is relatively stable in acidic or weakly acidic solutions, and unstable in neutral and slightly alkali solutions, which provides us an ideal tumor microenvironment sensitive theranostic nanoplatform (Scheme 1b). High quality SPECT images with zero background were successfully obtained in a subcutaneous 4T1 tumor model and deep tumors in critical locations, such as an orthotopic LM3 tumor model and *Mst1/2* double-knockout hepatoma model. The application of radiolabeled Pd nanosheets for photoacoustic (PA) imaging, and combined photothermal and radiotherapy was also successfully carried out.

## Results and discussion

### Synthesis and characterization

**Fig. 1:**
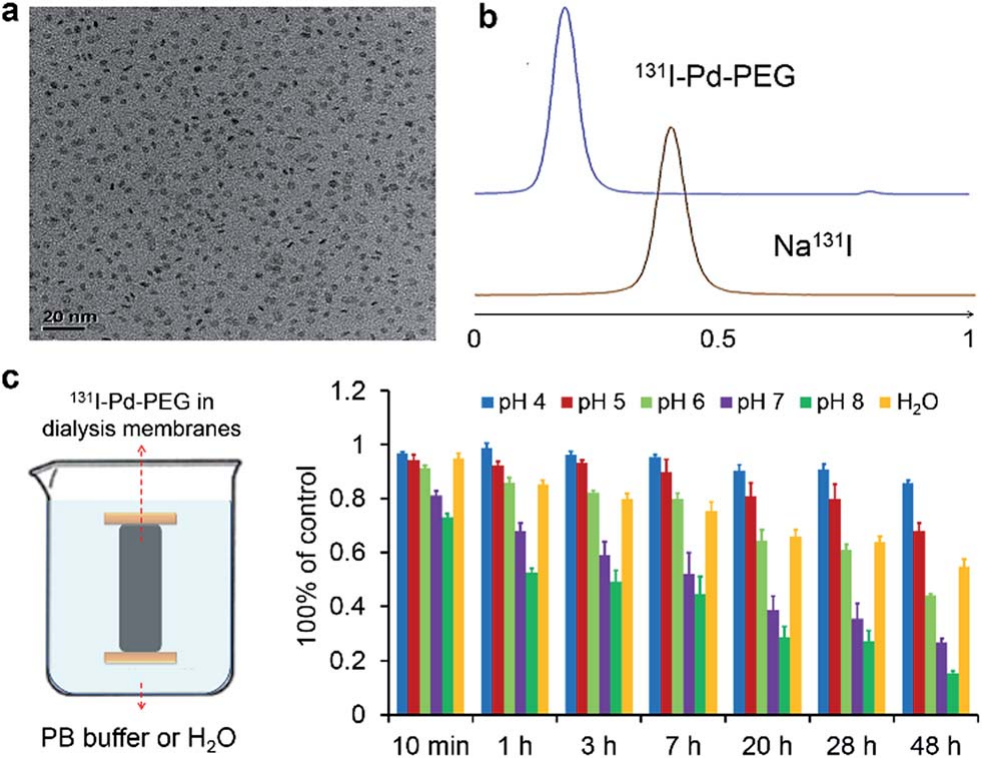
(a) Representative TEM image of Pd nanosheets. (b) TLC images of free ^131^I and ^131^I labeled Pd nanosheets. (c) Stability test of ^131^I–Pd–PEG in PB buffer with different pH values through dialysis.

Ultrasmall Pd nanosheets with an average size of 5 nm were synthesized according to our previous report (Fig. 1a).^14^ After that, Pd nanosheets were first modified with thiol-polyethylene glycol (mPEG-SH) to obtain PEGylated Pd nanosheets (Pd–PEG). Then, Pd–PEG was radiolabeled with Na^125^I or Na^131^I by simply stirring for 30 min at room temperature. The labeling yield was measured by ultrafiltration (Table S1†) and TLC (Fig. 1b). As expected, ^131^I labeled Pd–PEG (^131^I–Pd–PEG) showed a relatively high labeling efficiency (higher than 98%). Moreover, the TEM images of Pd nanosheets showed that there was no obvious change in morphology or size after treatment with different concentrations of NaI solution (Fig. S1†). This straightforward preparation procedure gives the radioiodine labeled probe a colossal competitive advantage in translational clinical research.

Interestingly, by tracking the radioactivity of radioiodine labeled Pd nanosheets, it was found that the adsorption of radioiodine on the surface of Pd nanosheets showed highly pH-dependent behavior. To better understand the behavior of radioiodine labeled Pd nanosheets in PB buffer with different pH values, dialysis bags (3500 Da, MWCO) were used to measure the desorption of radioiodine from ^131^I–Pd–PEG. Experiments showed that ^131^I–Pd–PEG exhibited good stability in acidic or weakly acidic solutions, and was unstable in neutral and slightly alkali solutions, which is caused by the coordinative competition between I^–^ and OH^–^ (Fig. 1c and S2†). As mentioned above, the microenvironment of the tumor is more acidic than that of the surrounding normal tissues. Therefore, we hypothesized that ^131^I–Pd–PEG is more stable in the tumor acidic microenvironment, which might contribute to high T/N ratios at the late stage of imaging studies. To the best of our knowledge, this is the first report on a pH-sensitive radioiodine labeled nanoplatform for tumor theranostics.

### SPECT/CT imaging of subcutaneous 4T1 tumor models

**Fig. 2:**
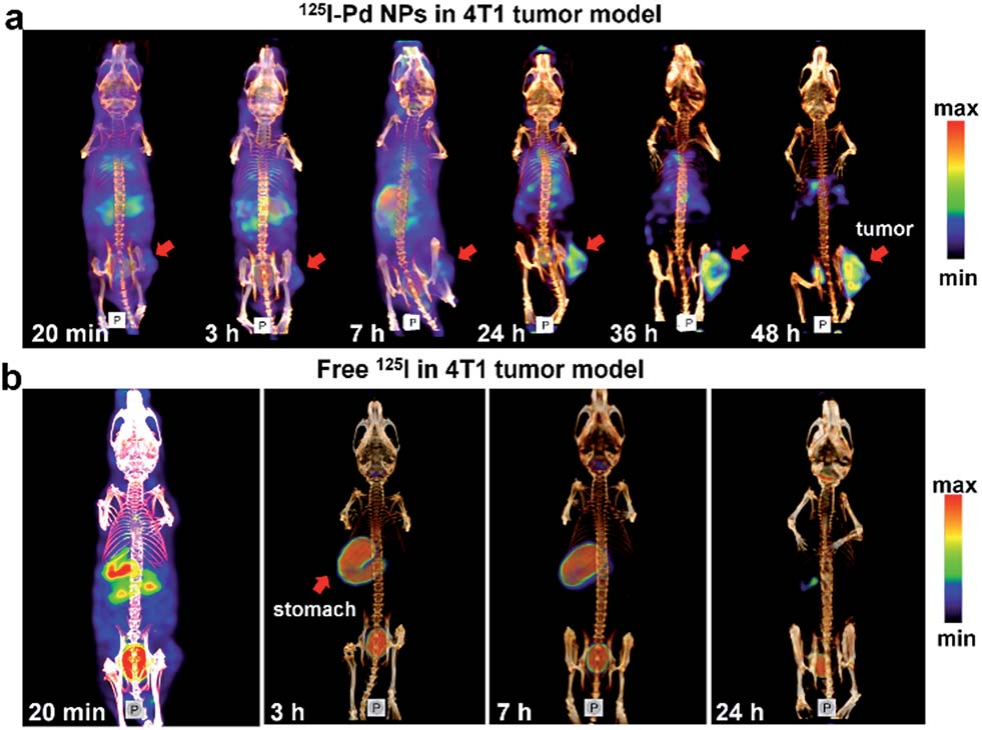
(a) SPECT/CT images of ^125^I–Pd–PEG in a subcutaneous 4T1 tumor model. (b) SPECT/CT images of Na^125^I in 4T1 tumor models at different times.

To test our hypothesis, SPECT imaging of ^125^I–Pd–PEG was performed with a micro-SPECT/CT scanner. Mice bearing subcutaneous 4T1 tumors were intravenously injected with ^125^I–Pd–PEG, and a significant uptake intensity was found in the blood, liver, lung and stomach at the initial imaging time (Fig. 2a). Pellucid tumor images could be achieved from 24 h p.i. to 48 h p.i. Meanwhile, nonspecific retentions of radioactivity in normal organs were decreasing and nearly negligible, which showed almost zero background. For comparison, SPECT imaging of Na^125^I in mice bearing subcutaneous 4T1 tumors was also assessed over time (Fig. 2b). Fast clearance of ^125^I from the heart and blood was observed and the tumor enhancement effect was not obvious.

To obtain more detailed information on the distribution of radioiodine labeled Pd nanosheets, biodistributions based on radioactivity of radioiodine and amount of Pd were studied. As expected, the distribution data of ^131^I–Pd–PEG *in vivo* measured with a γ-counter were well consistent with the SPECT images of ^125^I–Pd–PEG in mice bearing subcutaneous 4T1 tumors (Fig. S3a and b†). However, the distribution by radioactivity showed a different trend compared with that of Pd nanosheets measured by ICP-MS (Fig. S3c and d†). High radioactivity intensity was found in the stomach in the initial stage and the radioactivity in major organs and tissues was mostly cleared 18 h post injection of the ^131^I–Pd–PEG, while radioactivity in the tumor remained relatively stable, and thus increasing T/N ratios were obtained (Fig. S3b†). The ICP data showed that Pd nanosheets continued to accumulate in the tumor site, and there were obvious distributions in major organs (liver, lung, kidney and spleen). It's worth noting that the T/N ratios calculated from ICP data are relatively low compared with that by radioactivity. Moreover, the retention of ^131^I–Pd–PEG in blood also showed a similar trend as that of other normal organs and tissues when measured using a γ-counter and ICP-MS (Fig. S4 and S5†). All the above results further validate our hypothesis that ^131^I–Pd–PEG tends to be more stable in tumors than in normal organs or tissues. However, as the ^131^I was removed from ^131^I–Pd–PEG in the circulation, the radioactivity could not continue to accumulate in the tumor site as the Pd–PEG did. In this case, radioactivity in the tumor was not maximized. A possible way to solve this problem is to add target molecules on the surface of Pd nanosheets.

### SPECT/CT imaging of the orthotopic LM3 tumor model

**Fig. 3:**
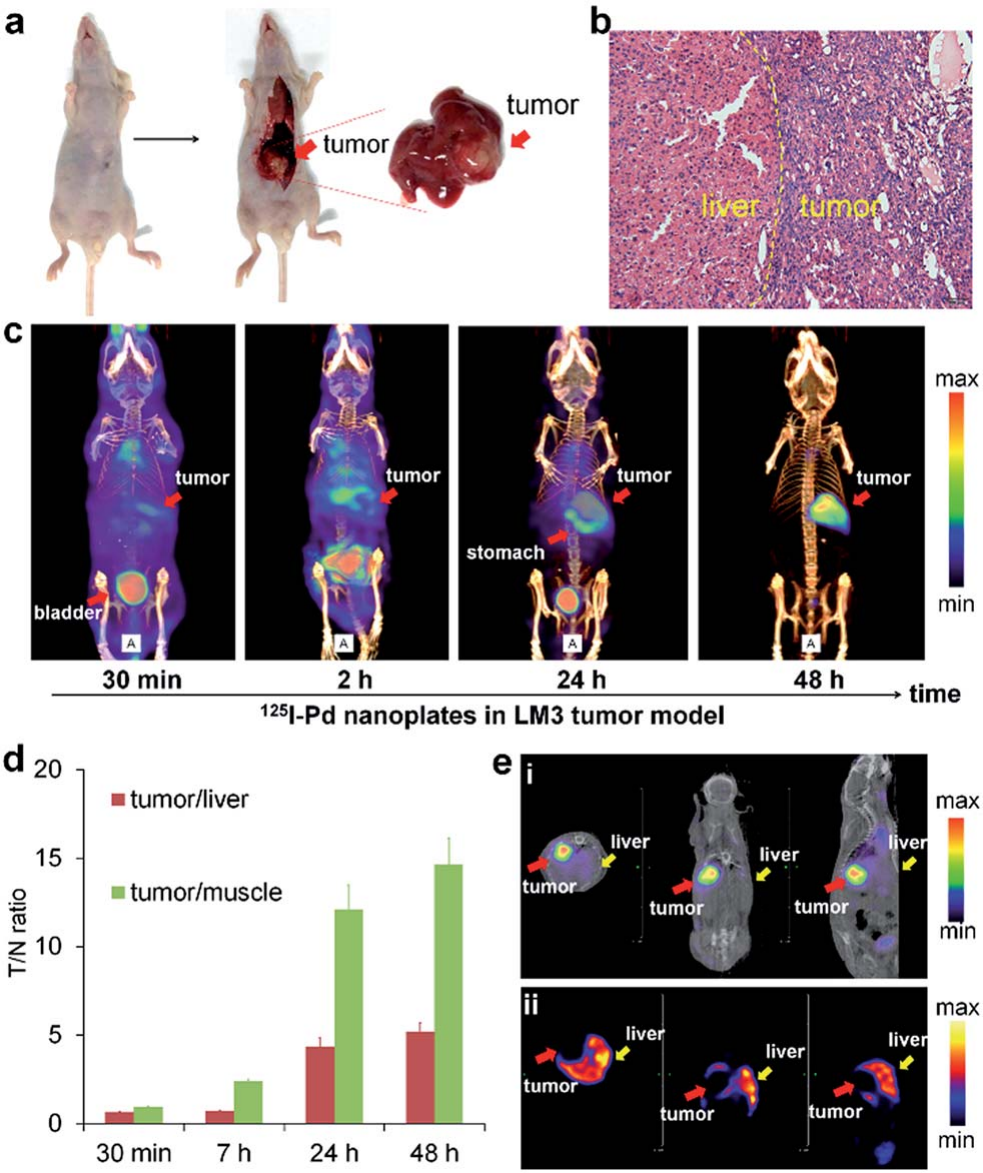
(a) Photographs of the liver from an LM3 tumor mouse after dissection. (b) Representative photomicrograph of H&E sections of the liver from the LM3 tumor mouse. (c) SPECT/CT images of ^125^I–Pd–PEG in the orthotopic LM3 tumor model at different times. (d) The radioactivity ratio of tumor to normal liver and muscle by calculating the radioactivity uptake in tumor, liver and muscle from SPECT images. (e) Simultaneous SPECT/CT images of ^125^I–Pd–PEG and ^99m^Tc-GSA in the ^125^I-window (i) and ^99m^Tc-window (ii), respectively.

The prominent T/N ratio makes us believe that radioiodine labeled Pd nanosheets can be used for diagnosis of deep tumors in critical locations. Experimentally, the LM3 tumor was inoculated orthotopically in mouse liver. Hematoxylin and eosin (H&E) staining was carried out to confirm the successful inoculation of the tumor model (Fig. 3a and b). From the SPECT imaging of ^125^I–Pd–PEG in mice bearing LM3 tumor, the radioactivity in tumor surrounding tissues was relatively high at the early stage and decreased obviously over time (Fig. 3c and S6†). At 48 h p.i., nearly no radioactivity signal was detected in the normal liver part and other normal organs or tissues, while still high radioactivity signal was found in the tumor, indicating that radioactivity remains stable in the deep tumor site and cleared faster from normal liver tissue. The result of autoradiography of the liver also provided another piece of evidence for the enhanced retention of radioiodine in the tumor (Fig. S7†). The time correlated radioactivity uptake of tumor, liver and muscle was calculated by drawing regions of interest (ROIs) on the SPECT images (Fig. S8†) and the T/N ratios showed an increasing trend post injection (Fig. 3d). For comparison, SPECT imaging of Na^125^I in orthotopic LM3 tumor-bearing mice was also assessed (Fig. S9†), and no obvious tumor enhancement effect was observed. To further confirm the tumor lesion imaged by ^125^I–Pd–PEG, a simultaneous dual-probe SPECT imaging strategy was proposed with the participation of ^99m^Tc-GSA (^99m^Tc labeled galactosyl human serum albumin) (Fig. 3e). ^99m^Tc-GSA is a radiolabeled liver-specific probe used for SPECT imaging of the ASGPR (asialoglycoprotein receptor). The normal part of the liver was readily delineated by SPECT images of ^99m^Tc-GSA. Interestingly, images of the LM3 tumor by ^125^I–Pd–PEG and the normal part of the liver could perfectly form a complete liver, suggesting that the radioiodine labeled Pd nanosheets are of immense potential in imaging of deep tumors.

### SPECT/CT imaging of the *Mst1/2* double-knockout hepatoma model

**Fig. 4:**
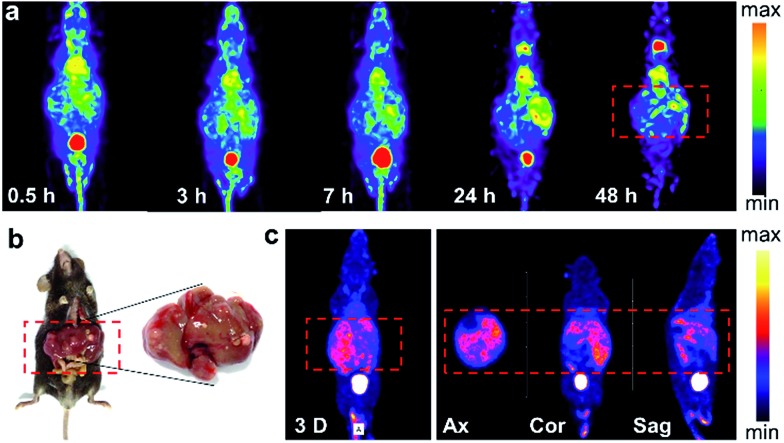
(a) SPECT images of ^125^I–Pd nanosheets in the *Mst1/2* double-knockout hepatoma model. (b) Photographs of liver tissue. (c) SPECT images of the *Mst1/2* DKO tumor model after i.v. injection with the ^99m^Tc-GSA probe.

The successful imaging of radioiodine labeled Pd nanosheets in both subcutaneous tumor and orthotropic liver tumor models motivated us to further explore more complex applications. Hence, in addition to the xenograft cancer model, a *Mst1/2* double-knockout-induced spontaneous hepatoma mouse model was introduced to test the versatility of ^125^I–Pd–PEG. As shown in the SPECT images (Fig. 4a), radioactivity in the whole mouse was detected at an early stage, and the tumor boundary became clear over time. Since many small primary tumors were found in the liver (Fig. 4b), the imaging of tumors was dispersive. Indeed, the non-uniform distribution of tumors in the liver was confirmed by using dissection and dual-isotope SPECT imaging. ^99m^Tc-GSA was used to differentiate tumor sites from pericarcinomatous tissue (Fig. 4c). With such outstanding sensitivity and specificity in tumors, ^125^I–Pd–PEG should be competent in the diagnosis of multiple types of cancer.

### PA imaging and combined cancer therapy

**Fig. 5:**
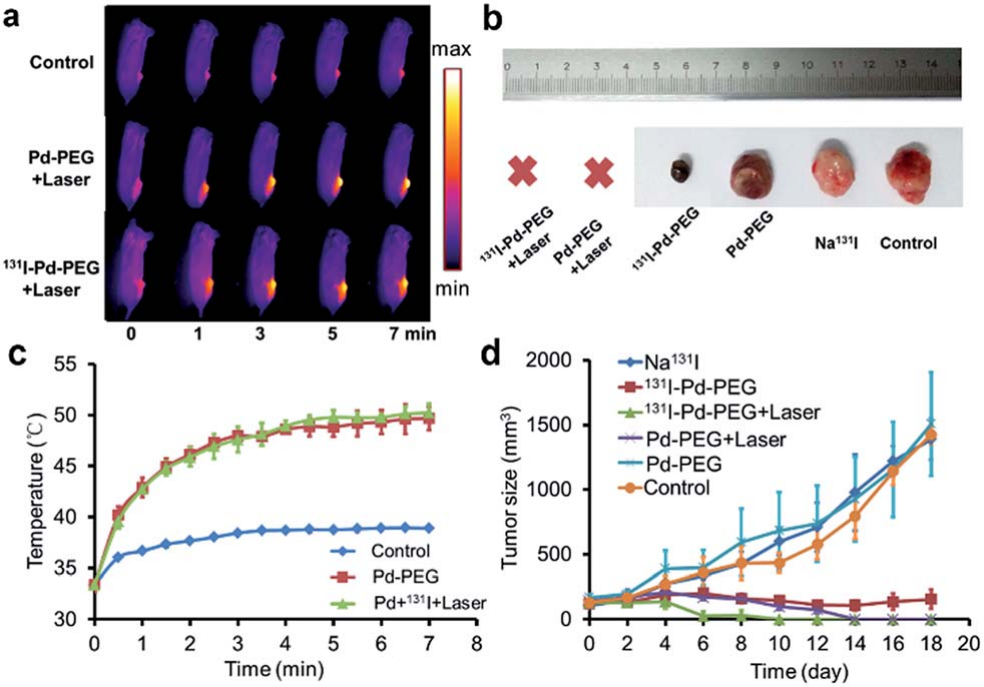
(a) IR images of tumor-bearing mice under irradiation from a 0.14 W cm^–2^ 808 nm laser. (b) Representative photographs of tumors after different treatments. (c) Temperature rise curves of tumor sites. (d) Time-dependent tumor growth curves of the 4T1 tumor (*n* = 10) under different treatments. Relative tumor volumes were normalized to their initial sizes.

Taking advantage of the excellent optical properties of Pd nanosheets, PA imaging was performed on a subcutaneous 4T1 tumor xenograft to confirm the successful tumor retention of Pd nanosheets, and test the multimodality imaging ability of our nanoplatform (Fig. S10†). As expected, a significantly enhanced PA signal was observed in tumors after injection of Pd nanosheets, further corroborating the accuracy of the SPECT/CT imaging results. Guided by the SPECT/CT and PA images, combined cancer therapy based on PTT and RT was performed. The photothermal effect of Pd nanosheets with different concentrations was first tested *in vitro* and the upward trend in temperatures was obvious at low concentrations (Fig. S11†). To verify the tumor-killing effect of ^131^I–Pd–PEG *in vivo*, 4T1 tumor-bearing mice were randomly divided into six groups with each group containing ten mice: PBS control group, Pd–PEG only, ^131^I only, ^131^I–Pd–PEG only, Pd–PEG + laser, and ^131^I–Pd–PEG + laser. Groups with laser irradiation were subjected to 808 nm laser treatment at an ultra-low power density of 0.14 W cm^–2^, and an infrared thermal camera was used to monitor the temperature changes of tumor sites (Fig. 5a). A quick temperature rise was detected in Pd–PEG + laser and ^131^I–Pd–PEG + laser groups (Fig. 5c). The tumor sizes were measured using a caliper every other day after treatment. Improved therapeutic efficacy was observed in mice injected with ^131^I–Pd–PEG in delaying the tumor growth, while the mice treated with free ^131^I showed a growth trend similar to that of the control group (Fig. 5b and d). Excitingly, after receiving combined RT and PTT, tumors of mice treated with ^131^I–Pd–PEG + laser were significantly damaged and showed an enhanced inhibition effect on tumor growth compared with mice treated with Pd–PEG + laser, confirming the remarkable *in vivo* synergistic anti-tumor therapeutic effect of our combination therapy by ^131^I–Pd–PEG. ^18^F-FDG PET/CT was used to further confirm the therapeutic efficacy of ^131^I + Pd and ^131^I + Pd + laser groups and no tumor regrowth was detected in mice treated with ^131^I–Pd–PEG (Fig. S12†). During the treatment of each group, we did not notice any obvious sign of toxic side effects and neither death nor a significant body weight drop was noted (Fig. S13†). The major organs of ^131^I–Pd–PEG + laser treated mice whose tumors were eliminated by the photothermal therapy were collected 18 days after the treatment for histology analysis. No noticeable signal of organ damage was observed from H&E stained organ slices (Fig. S14†).

## Conclusions

In summary, we report a successful radiolabeling of ultrasmall Pd nanosheets utilizing the specific adsorption of halide ions on the surface of Pd nanosheets. With the excellent labeling efficiency achieved under mild conditions, the simple radiolabeling processes reported in this work show great clinical translation potential. The accumulation of ^131^I–Pd–PEG was mainly attributed to the EPR effect and phagocytosis of cancer cells. As a pH-sensitive radiolabeled theranostic nanoagent, the radioiodine labeled on the surface of Pd nanosheets was more stable in an acidic environment, and thus showed a stable retention of radioisotopes in tumor sites. In the SPECT imaging study, ^125^I–Pd–PEG exhibited a significantly high T/N ratio in the subcutaneous 4T1 tumor xenograft and orthotopic HCC mouse model. To mimic the actual conditions, *Mst1/2* DKO tumor models were established to evaluate the general applicability of ^125^I–Pd–PEG and high quality SPECT images with a zero background signal of tumors were achieved. Enhanced tumor retention of Pd nanosheets was also confirmed by PA imaging. Moreover, ^131^I–Pd–PEG readily served as a therapeutic platform for the combination of photothermal therapy and internal radiotherapy in cancer treatment, and achieved a remarkable synergistic effect in killing cancer. This study provides important guidelines for future research on radiochemistry and *in vivo* bioapplications of nanomaterials. More studies are still needed to develop effective strategies to allow fast and high accumulation of ^125^I–Pd–PEG in tumor sites.

## Experimental section

### Reagents and instruments

Na^131^I and Na^99m^TcO_4_ were obtained from Zhongshan Hospital Affiliated of Xiamen University. The ^18^F-FDG was obtained from the First Affiliated Hospital of Xiamen University. Na^125^I was obtained from China Isotope & Radiation Corporation. GSA kits were obtained from Beijing Shihong Pharmaceutical Center of Beijing Normal University. Palladium(ii) acetylacetonate (Pd(acac)_2_, 99%) was bought from Alfa Aesar. Poly(vinylpyrrolidone) (PVP K30) was obtained from Sinopharm Chemical Reagent Co. Ltd. (Shanghai, China). Methoxypoly(ethylene glycol) thiol (mPEG-SH, 5 K) was bought from Sinopeg Biotech Co., Ltd. *N*,*N*-Dimethylpropionamide (DMP) was obtained from Sigma-Aldrich Co., LLC. The TLC strips were detected with a Mini-Scan radio-TLC Scanner (BioScan, USA). The radioactivity was measured with a γ-counter (WIZARD 2480, Perkin-Elmer, USA) and CRC-25R Dose Calibrators (CAPIN-TEC. Inc, USA). The SPECT imaging study was performed with a nanoScan SPECT/CT scanner (Mediso, HUNGARY). Animal PET/CT scans were performed using an Inveon device (Siemens Medical Solutions Inc., USA). PA imaging was performed with Nexus 128 photoacoustic tomography systems (Ann Arbor, MI, USA). TEM images were recorded on a TECNAI F-30 high-resolution transmission electron microscope operating at 300 kV.

### Animal experiments

All the mice were obtained from the Laboratory Animal Center of Xiamen University. All animal procedures were in accordance with the National Institute of Health Guidelines for the Care and Use of Laboratory Animals and were approved by the Animal Ethics Committee of Xiamen University.

Balb/c mice bearing 4T1 murine breast cancer tumors, nude mice bearing HCC-LM3 human hepatocarcinoma and gene knockout mice models bearing liver tumors were used in this study. The 4T1 murine breast tumor models were generated by subcutaneous injection of 5 × 10^6^ cells (in 50 μL PBS) into the right rear flanks of each mouse (female Balb/c mouse) and the consequent tumor was allowed to grow for 7 days.

To establish the orthotopic HCC mouse model, each nude mouse (male, 4–5 weeks) was implanted with 5 × 10^6^ human hepatocellular carcinoma HCC-LM3 cells through surgery and the consequent tumor was allowed to grow for 2 weeks.

*Mst1/2* DKO tumor models were provided by Professor Dawang Zhou from Xiamen University. Targeted ES clones were microinjected into C57BL/6 blastocysts. *Mst1^fl/fl^ Mst2^fl/fl^* chimeric offspring were crossed to Alb-cre mice to generate mice with *Mst1* and *Mst2* mutants in the liver. Hepatomegaly and hepatoma could be observed in *Mst1^fl/fl^ Mst2^fl/fl^* Alb-cre mice within three months. For details, see ref. 15.

### Preparation of 5 nm Pd nanosheets

10.0 mg of Pd(acac)_2_, 32.0 mg of PVP, and 30 mg of NaBr were mixed together with 2 mL of *N*,*N*-dimethylpropionamide and 4 mL of water in a 48 mL glass pressure vessel. The vessel was then charged with CO to 1 bar and heated from room temperature to 100 °C in 0.5 h, and then kept at 100 °C for another 2.5 h. The obtained product was stored at 4 °C for further use.

### Surface PEGylation of Pd nanosheets

1 mg of Pd nanosheets was first precipitated with acetone then redispersed in 1 mL of mPEG-SH aqueous solution (20 mg mL^–1^). The mixtures were stirred for 30 min at room temperature, and then kept in a refrigerator overnight. Free mPEG-SH was removed by ultrafiltration before use.

### Radiolabeling procedure

Pd nanosheets were radiolabeled with ^125/131^I by simply stirring. 100 μL of Na^125/131^I in H_2_O was added to solution of Pd nanosheets, and the resulting solution was stirred for 30 min at room temperature. The labeling yield was measured by centrifugation (10 000 rpm for 10 min, repeated 3 times) and TLC (polyamide film/saline).

### Stability test

A dialysis bag (3500 Da MWCO) was used to investigate the stability in PB buffer under various pH conditions. The dialysate was changed every 12 hours. The radioactivity counts in the dialysis membranes were measured with a γ-counter at 10 min, 1 h, 3 h, 7 h, 20 h, 28 h, and 48 h.

### SPECT imaging

The feasibility of micro-SPECT/CT imaging with ^125^I–Pd–PEG for tumor detection was investigated in several kinds of tumor models (subcutaneous 4T1 tumor model, orthotopic LM3 tumor model and *Mst1/2* double-knockout hepatoma model). ^125^I–Pd–PEG (37 MBq/200 μL, 10 mg kg^–1^) was administered into each mouse through tail vein injection. The tumor uptake was determined by selecting the region of interest (ROI) and comparing it with liver and muscle tissues. To correctly locate the tumor site and liver outline, ^99m^Tc-GSA was used in the SPECT imaging study for proper comparison with ^125^I–Pd. The acquiring parameters were as follows: energy peak of 140.5 keV for ^99m^Tc and 28 keV for ^125^I, window width of 20%, matrix of 256 × 256, medium zoom, and frame: 30 s.

4T1 tumor mice and LM3 tumor mice were used for the investigation of the biodistribution of free ^125^I. Na^125^I (37 MBq/200 μL in saline) was administered into each mouse through tail vein injection. SPECT imaging for 4T1 tumor mice was performed at 15 min, 3 h, 7 h and 24 h after injection of Na^125^I. For LM3 tumor models, the imaging time points were 30 min, 2 h and 24 h.

### Radio-biodistribution

4T1 tumor models were used in the biodistribution study. ^131^I–Pd–PEG (1.85 MBq/200 μL, 10 mg kg^–1^) was administered into each mouse through tail vein injection. The mice were sacrificed at different time points. Radioactivity of major organs and tumors was measured with a γ-counter. The results were shown as a percentage of the injected dose per gram of tissue (% ID per g).

### ICP-MS test

Inductively coupled plasma mass spectrometry (ICP-MS, Agilent 7700x) was used to obtain quantitative measurement of the distribution of Pd nanosheets. After radio-biodistribution characterization, major organs and tumors were weighed and digested using HNO_3_ and H_2_O_2_ (volume ratio 4 : 1). The results were shown as a percentage of the injected dose per gram of tissue (% ID per g).

### Autoradiography study

After SPECT imaging of ^125^I–Pd–PEG in a nude mouse bearing the HCC-LM3 tumor, the mouse was sacrificed and the liver was harvested for an autoradiography study.

### PA imaging

PA images of Pd nanosheets with concentrations of 0.0155, 0.031, 0.0625, 0.125, 0.25, 0.5, and 1.0 mg mL^–1^ were recorded in tubes. Mice bearing the 4T1 tumor on the right back were i.v. injected with 200 μL of Pd–PEG (1 mg mL^–1^). PA images were collected using a Nexus 128 scanner (Ann Arbor, MI, USA) with an 808 nm working laser before and 0.5, 2, 4, 7, 24, and 48 h after i.v. injection.

### Radiotherapy (RT) and photothermal therapy (PTT)

200 uL of ^131^I–Pd–PEG were intravenous injected into mice bearing 4T1 tumors. 24 h later, the tumors were irradiated with an 808 nm laser at an ultra-low power density of 0.14 W cm^–2^, and an infrared (IR) thermal camera was used to monitor the temperature changes of the tumor sites. The radiotherapy was autostarted just after the injection of ^131^I–Pd–PEG. The tumor size and mouse weight were measured every 2 days after treatment. PET/CT was used to monitor the therapeutic efficacy of ^131^I + Pd and ^131^I + Pd + laser groups with the help of ^18^F-FDG (about 100 μCi per mouse).

### Histology examination

The acute toxicity of ^131^I–Pd–PEG was assessed on normal mice and the ^131^I + Pd + laser group. Major organs were excised and histological analysis was performed on the mice 40 days after radiotherapy and photothermal therapy. The tissues of the treatment group showed a similar histological structure to the normal mice.

## Conflicts of interest

There are no conflicts to declare.

## Supplementary Material

Supplementary information
