# Supplementary material for: Pd nanosheets with their surface coordinated by radioactive iodide as a high-performance theranostic nanoagent for orthotopic hepatocellular carcinoma imaging and cancer therapy
Source: Chem Sci. 2018 Apr 12;9(18):4268–74. doi: 10.1039/c8sc00104a (PMC5944209; doi:10.1039/c8sc00104a)
Supplement: Supplementary file 1 [file SC-009-C8SC00104A-s001.pdf]

Electronic Supplementary Material (ESI) for Chemical Science.
This journal is © The Royal Society of Chemistry 2018

Supporting Information

Pd Nanosheets with Surface Coordinated by Radioactive Iodide as a High-Performance Theranostic Nanoagent for Orthotopic Hepatocellular Carcinoma Imaging and Cancer Therapy

Mei Chen, [#ab] *Zhide Guo,* [#c] *Qinghua Chen,*[d] *Jingping Wei,*[b] *Jingchao Li,*[b]*Changrong Shi,*[c] *DuoXu,*[c] *Dawang Zhou,*[d] *Xianzhong Zhang,**[d] *and Nanfeng Zheng**[b]

Contents:

Table S1. Labeling efficiency of radioactive iodine (^{131}I) on the surface of Pd nanosheets.

Figure S1. TEM images of Pd nanosheets after labeled with different amount of NaI.

Figure S2. Stability test of ^{131}I-Pd-PEG in BSA-PB buffer with different pH values through dialysis.

Figure S3. (a) Biodistribution of ^{131}I-Pd-PEG in mice bearing 4T1 tumor at different time points measured by γ-counter. (b) T/N ratios calculated from the biodistribution data. (c) Biodistribution of Pd nanosheets in mice bearing 4T1 tumor at different time points measured by ICP method. (d) T/N ratios calculated from the ICP data.

Figure S4. Blood circulation histogram of ^{131}I-Pd-PEG determined by measuring the radioactivity in blood at different time.

Figure S5. Blood circulation histogram of ^{131}I-Pd-PEG determined by measuring the Pd amount in blood at different time.

Figure S6. SPECT/CT images of ^{125}I-Pd-PEG in LM3 tumor models.

Figure S7. Autoradiography of liver and tumor specimen.

Figure S8. Radioactivity uptake in tumor, liver and muscle calculated from SPECT images.

Figure S9. SPECT/CT images of Na^{125}I in LM3 tumor models.

Figure S10. (a) PA imaging of Pd nanosheets with different concentrations. (b) Quantification PA signals of Pd nanosheets with different concentrations. (c) PA imaging of Pd nanosheets in 4T1 tumor models. (d) Quantification PA signals of tumor site at different time post i.v. injection.

Figure S11. (a) Photothermal effect of Pd nanosheets with different concentrations. (b) Temperature rise curves of Pd nanosheets with different concentrations.

Figure S12. ^{18}F-FDG PET/CT images of normal mice and mice bearing 4T1 tumor under different treatments.

Figure S13. Body weight curves of different groups.

Figure S14. Representative photomicrographs of H&E sections of major organs from normal mice and ^{131}I+Pd+laser group after treatment.

Table S1. Labeling efficiency of radioactive iodine (^{131}I) on the surface of Pd nanosheets.

material mass	activity	centrifugation		
		1 st	2 nd	3 rd
200 µg Pd nanosheets	1.85 MBq ^{131}I	100%	100%	98%
200 µg Pd nanosheets	3.7 MBq ^{131}I	100%	100%	98%
200 µg Pd nanosheets	9.25 MBq ^{131}I	99%	98%	98%
200 µg Pd nanosheets	27.75 MBq ^{131}I	100%	99%	98%
200 µg Pd nanosheets	55.5 MBq ^{131}I	100%	99%	98%

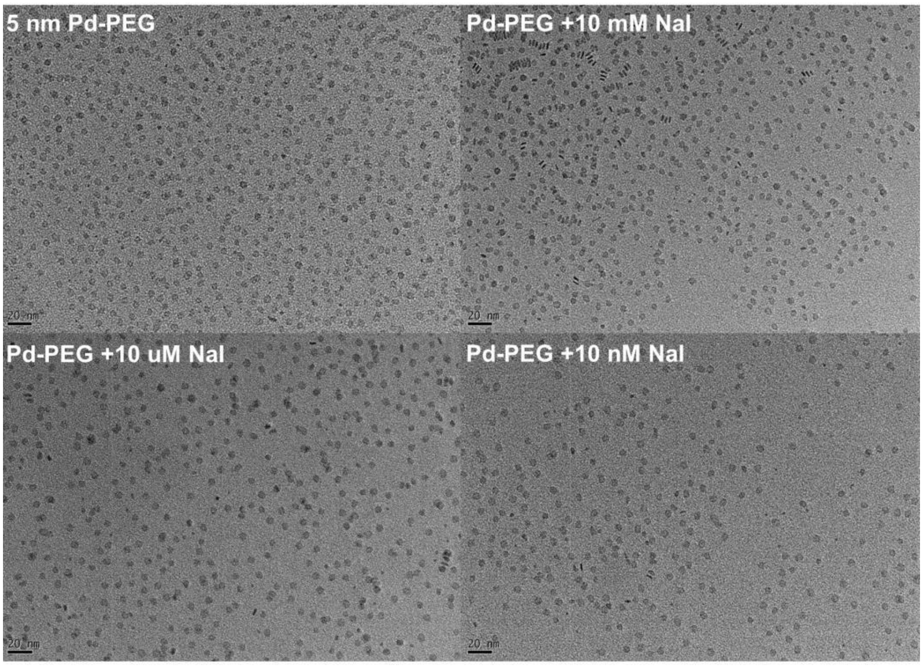

Figure S1. TEM images of Pd nanosheets after labeled with different amount of NaI.

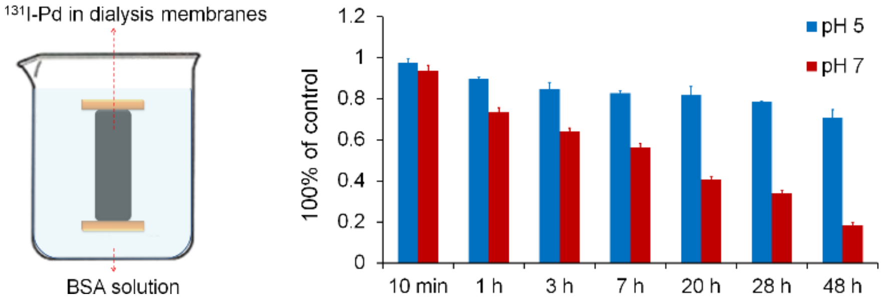

Figure S2. Stability test of ^{131}I-Pd-PEG in BSA-PB buffer with different pH values through dialysis.

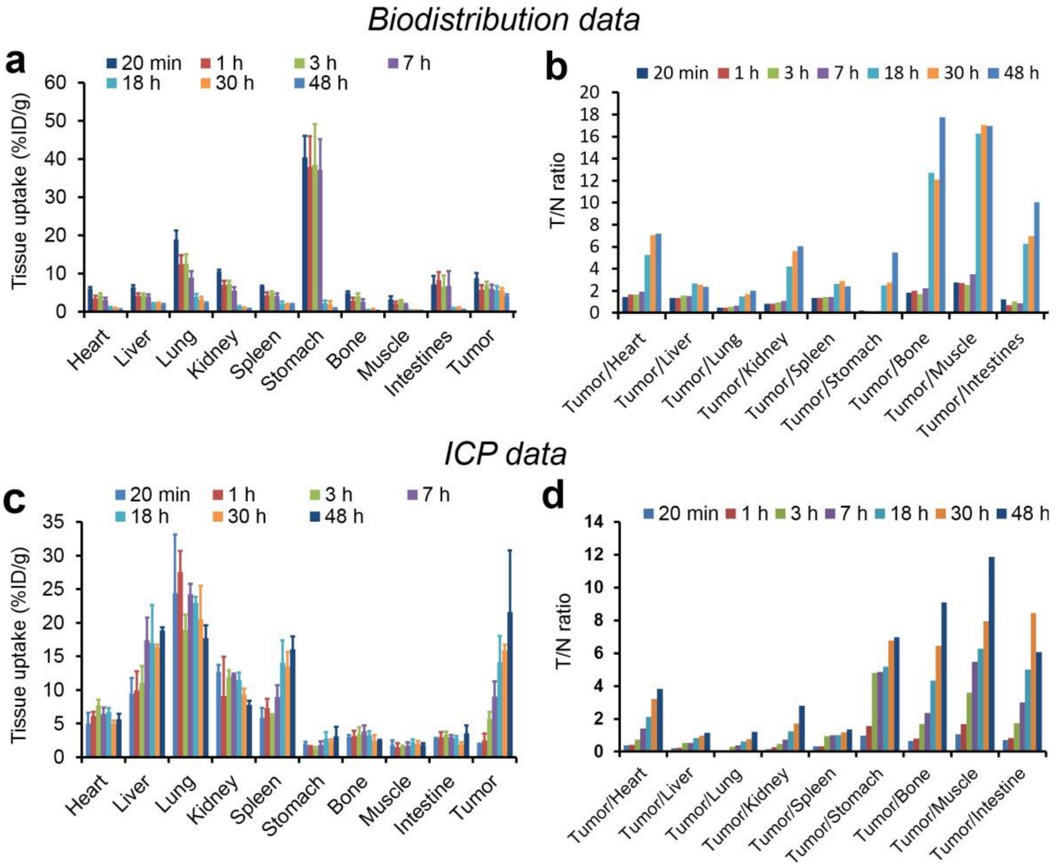

Figure S3. (a) Biodistribution of ^{131}I in 4T1 tumor bearing mice at deffrernt time points measured by γ-counter. (b) T/N ratios calculated from the biodistribution data. (c) Biodistribution of Pd nanosheets in 4T1 tumor bearing mice at deffrernt time points measured by ICP-MS method. (d) T/N ratios calculated from the ICP data.

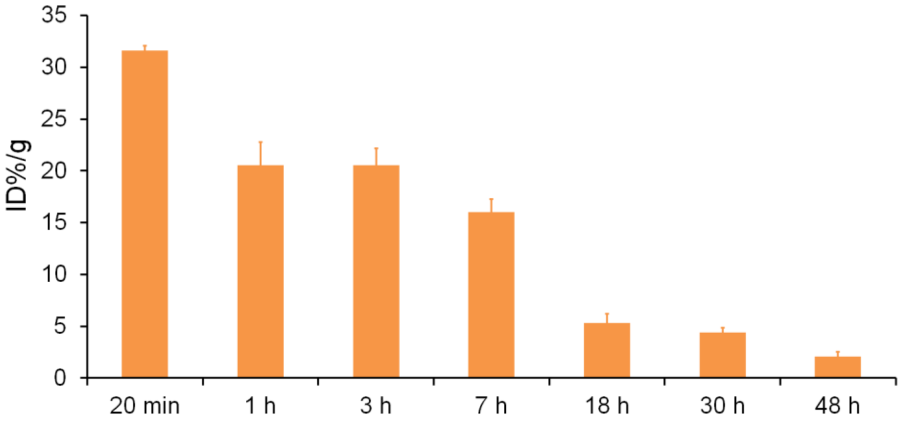

Figure S4. Blood circulation histogram of ^{131}I-Pd-PEG determined by measuring the radioactivity in blood at different time.

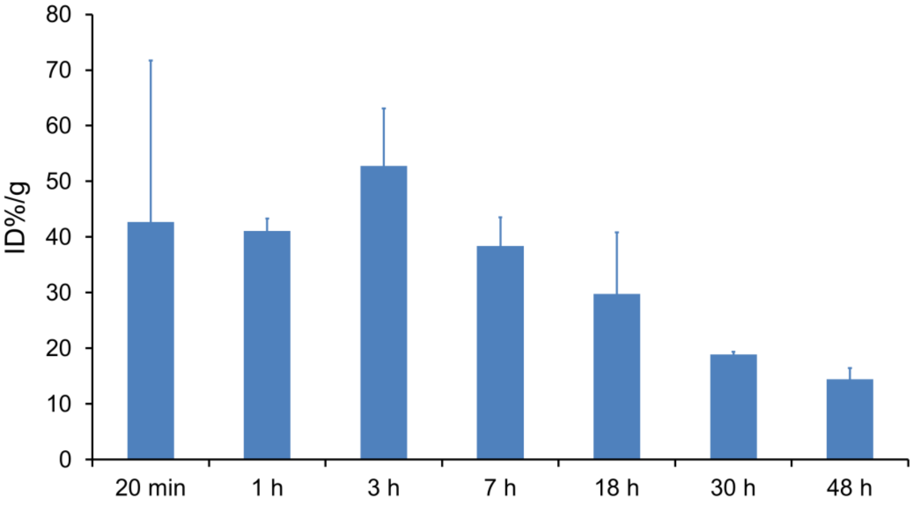

Figure S5. Blood circulation histogram of ^{131}I-Pd-PEG determined by measuring the Pd amount in blood at different time.

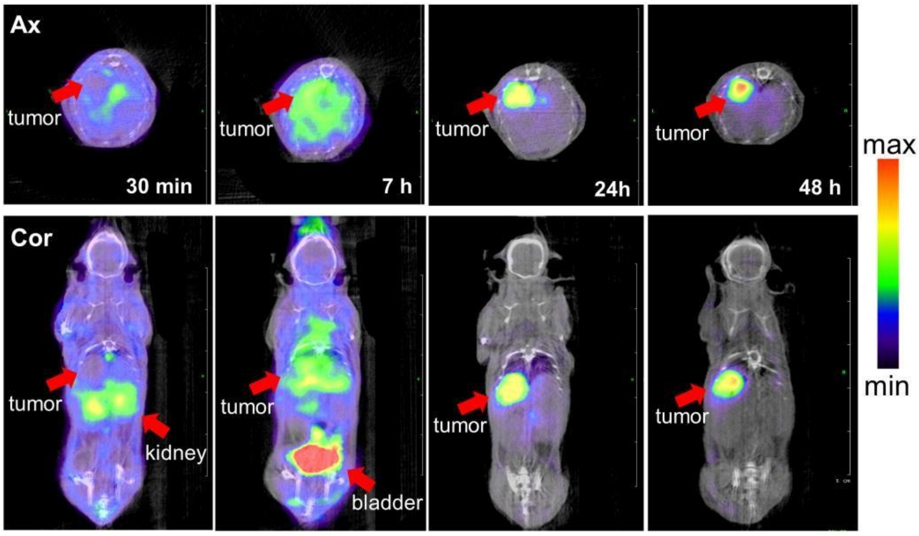

Figure S6. SPECT/CT images of ^{125}I-Pd-PEG in LM3 tumor models.

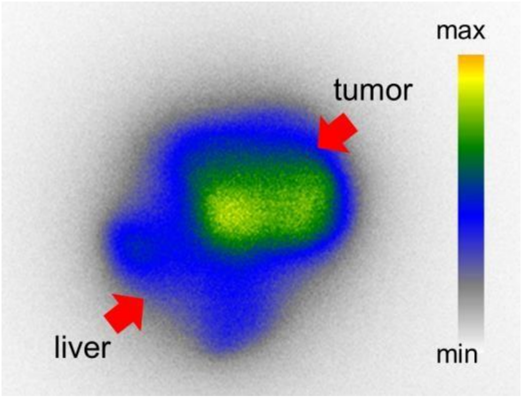

Figure S7. Autoradiography of liver and tumor specimen.

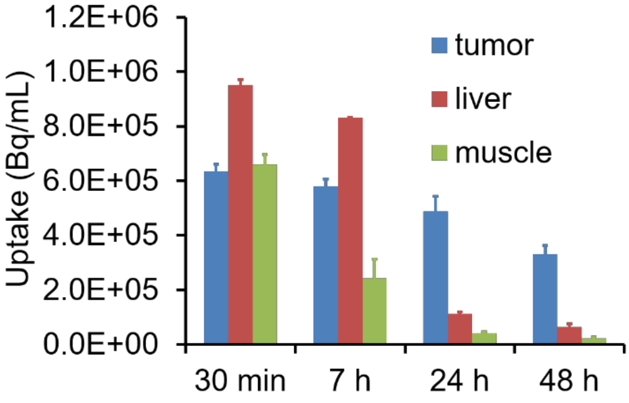

Figure S8. Radioactivity uptake in tumor, liver and muscle calculated from SPECT images.

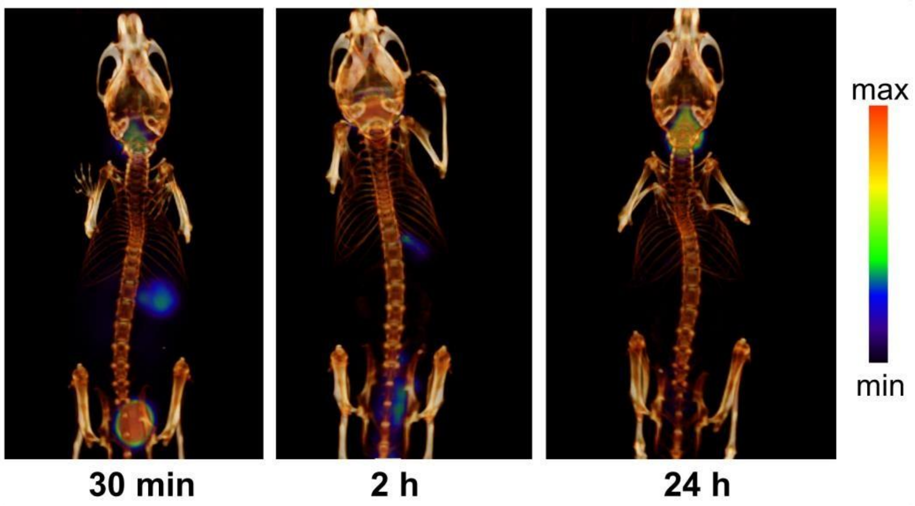

Figure S9. SPECT/CT images of $Na^{125}I$ in LM3 tumor models.

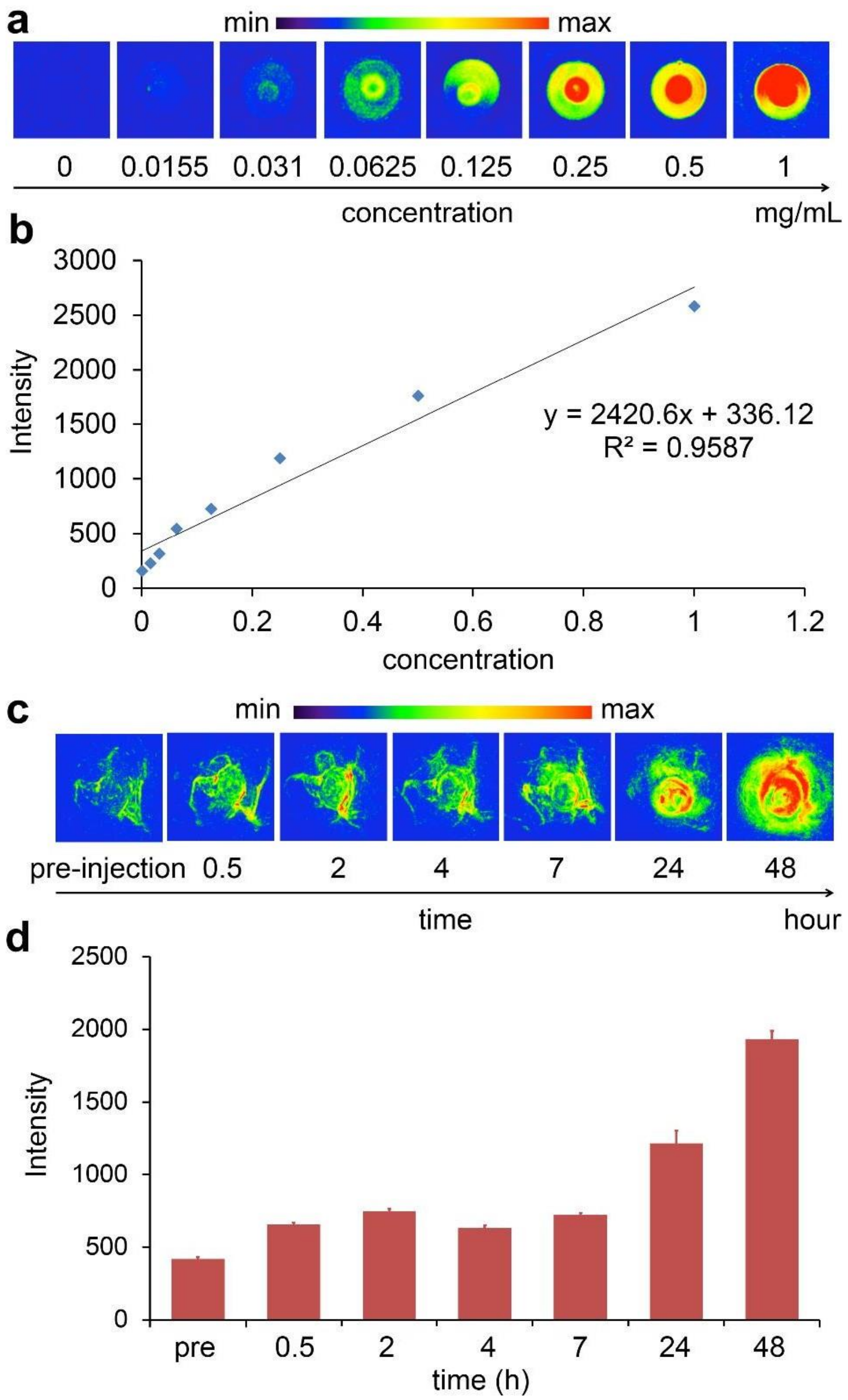

Figure S10. (a) PA imaging of Pd nanosheets with different concentrations. (b) Quantification PA signals of Pd nanosheets with different concentrations. (c) PA imaging of Pd nanosheets in 4T1 tumor models. (d) Quantification PA signals of tumor site at different time post i.v. injection.

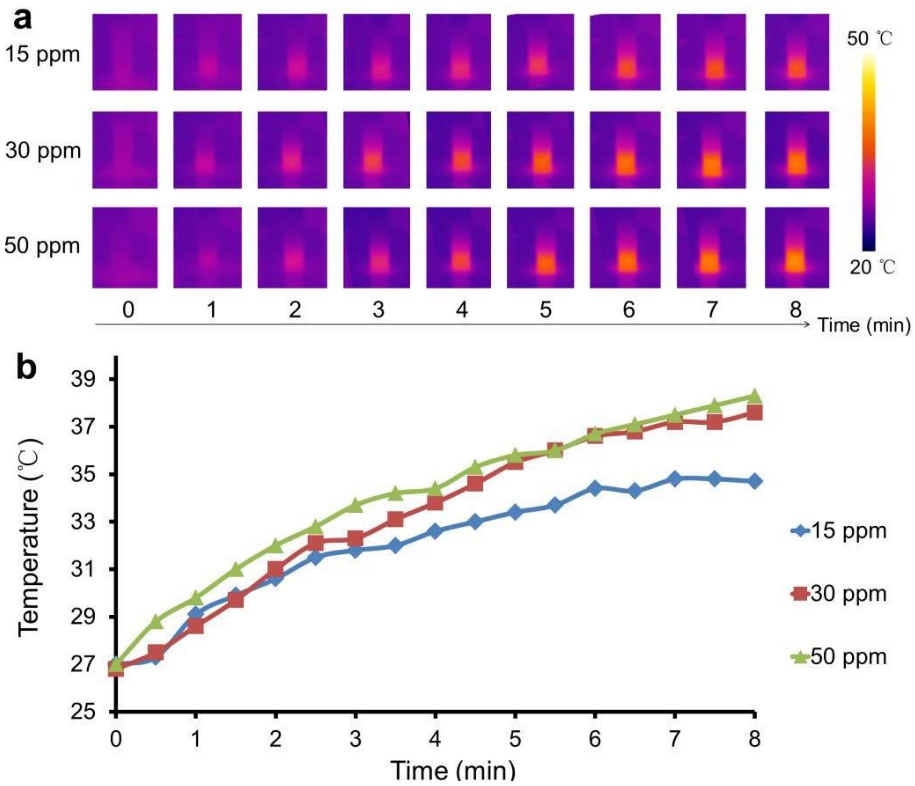

Figure S11. (a) Photothermal effect of Pd nanosheets with different concentrations. (b)Temperature rise curves of Pd nanosheets with different concentrations.

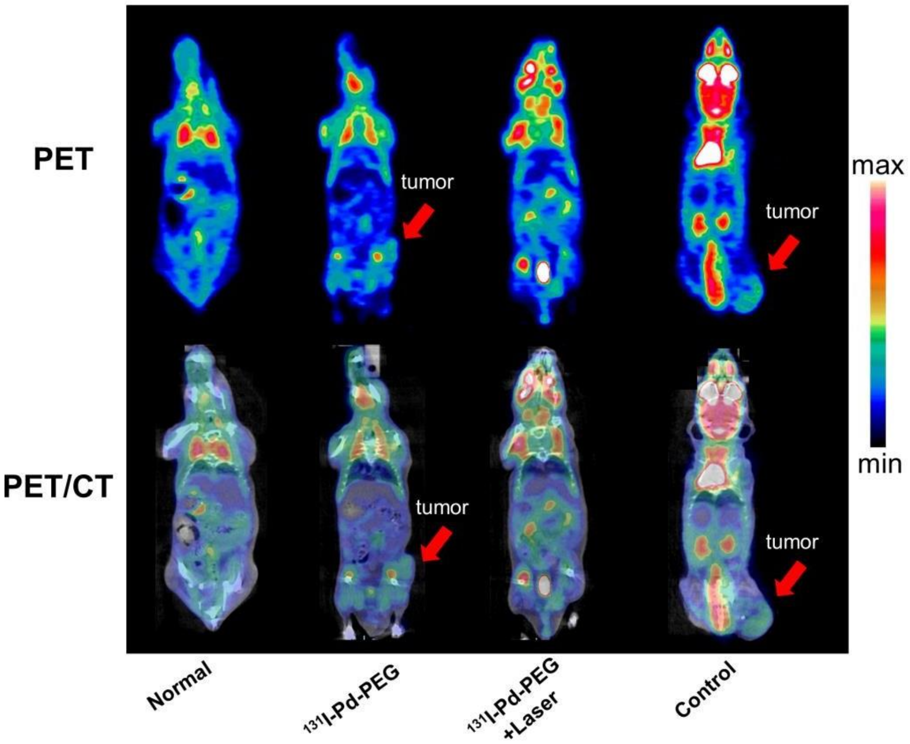

Figure S12. ^{18}F-FDG PET/CT images of normal mice and 4T1 tumor bearing mice under different treatments.

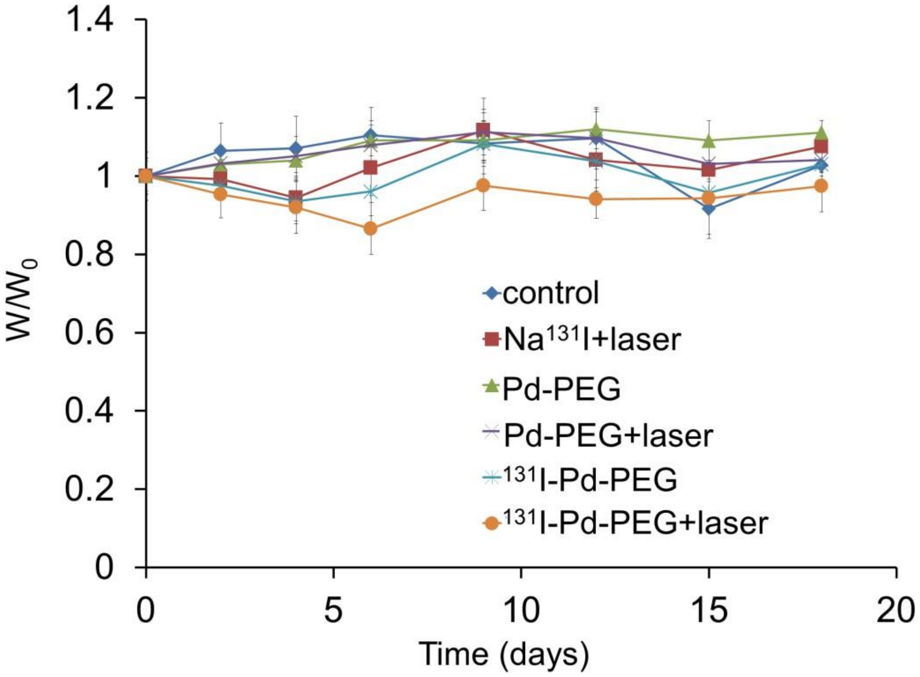

Figure S13. Body weight curves of different groups.

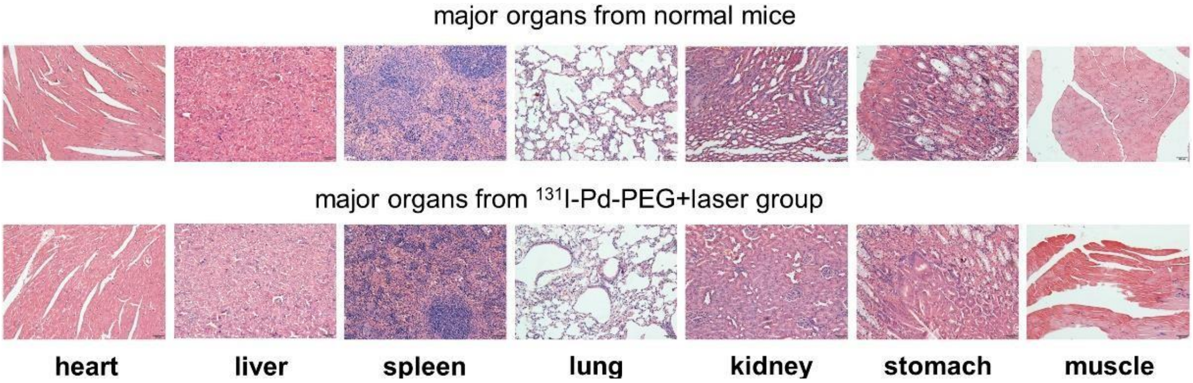

Figure S14. Representative photomicrographs of H&E sections of major organs from normal mice and ^{131}I+Pd+laser group after treatment.
